# Supplementary material for: Novel Mn4+-Activated K2Nb1−xMoxF7 (0 ≤ x ≤ 0.15) Solid Solution Red Phosphors with Superior Moisture Resistance and Good Thermal Stability
Source: Molecules. 2023 Jun 5;28(11):4566. doi: 10.3390/molecules28114566 (PMC10254550; doi:10.3390/molecules28114566)
Supplement: Supplementary file 1 [file molecules-28-04566-s001.zip › molecules-2371105-supplementary.pdf]

Supplementary Materials

Novel Mn^{4+}-Activated $K_2Nb_{1-x}Mo_xF_7$ ($0 \le x \le 0.15$) Solid Solution Red Phosphors with Superior Moisture Resistance and Good Thermal Stability

Yuhan Gao, Lei Feng, Linglin Wang, Jun Zheng, Feiyao Ren, Siyu Liu, Zhanglei Ning *, Ting Zhou, Xiaochun Wu, Xin Lai and Daojiang Gao *

College of Chemistry and Materials Science, Sichuan Normal University, Chengdu 610066, China; 2020080608@stu.sicnu.edu.cn (Y.G.); 20201201055@stu.sicnu.edu.cn (L.F.); 20221201058@stu.sicnu.edu.cn (L.W.); 2020080243@stu.sicnu.edu.cn (J.Z.); 2020080221@stu.sicnu.edu.cn (F.R.); 2020080622@stu.sicnu.edu.cn (S.L.); ting_zhou@sicnu.edu.cn (T.Z.); wuxiaochun@sicnu.edu.cn (X.W.); laixin1972@sicnu.edu.cn (X.L.)

* Correspondence: zlning@sicnu.edu.cn (Z.N.); daojianggao@sicnu.edu.cn (D.G.)

List of Figures

Figure S1. XRD Rietveld refinement patterns of $K_2Nb_{1-x}Mo_xF_7$: Mn^{4+} samples. S4

Figure S2. Diffuse reflection spectra of K_2NbF_7:Mn^{4+} (KNF) (a) and $K_2Nb_{1-x}Mo_xF_7$: Mn^{4+} with $x = 0.05$ (KNMF) (c); band gaps of KNF (b) and KNMF (d). S5

Figure S3． Quantum yields of K_2NbF_7:Mn^{4+} (KNF) (a) and $K_2Nb_{1-x}Mo_xF_7$: Mn^{4+} with $x = 0.05$ (KNMF) (b). S6

Figure S4. Schematic diagram of the preparation process for K_2MnF_6 and $K_2Nb_{1-x}Mo_xF_7$:Mn^{4+} phosphors. S7

List of Tables

Table S1. Fitting parameters of decay lifetimes for the $K_2Nb_{1-x}Mo_xF_7$: Mn^{4+} phosphors........... S9

Figure S1. XRD Rietveld refinement patterns of $K_2Nb_{1-x}Mo_xF_7$: Mn^{4+} samples.

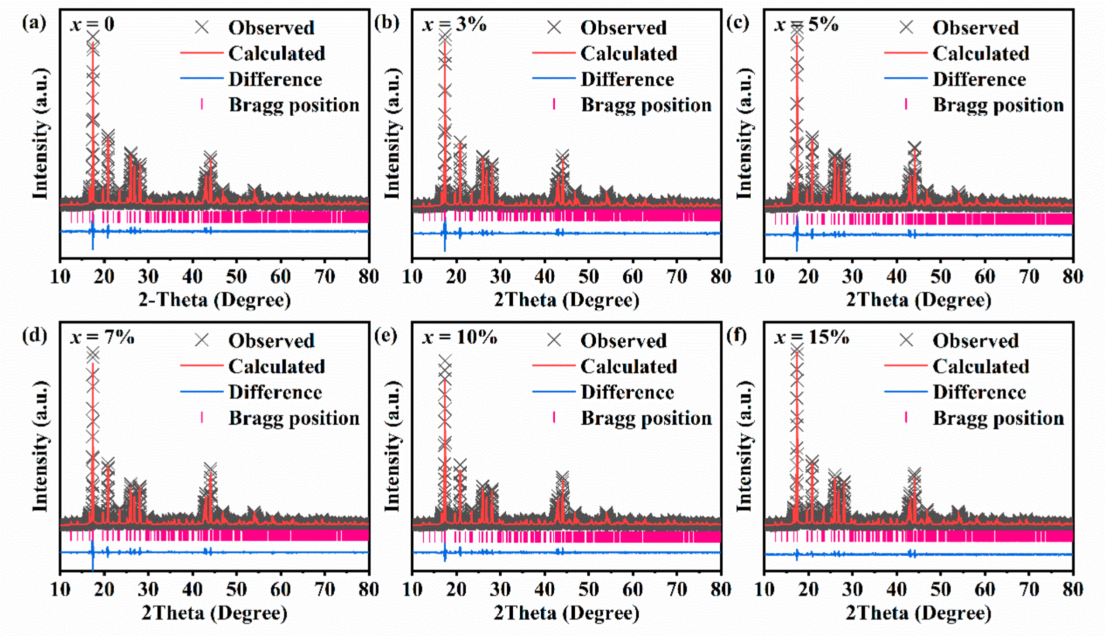

Figure S2. Diffuse reflection spectra of K_2NbF_7:Mn^{4+} (KNF) (a) and $K_2Nb_{1-x}Mo_xF_7$: Mn^{4+} with x = 0.05 (KNMF) (c); band gaps of KNF (b) and KNMF (d).

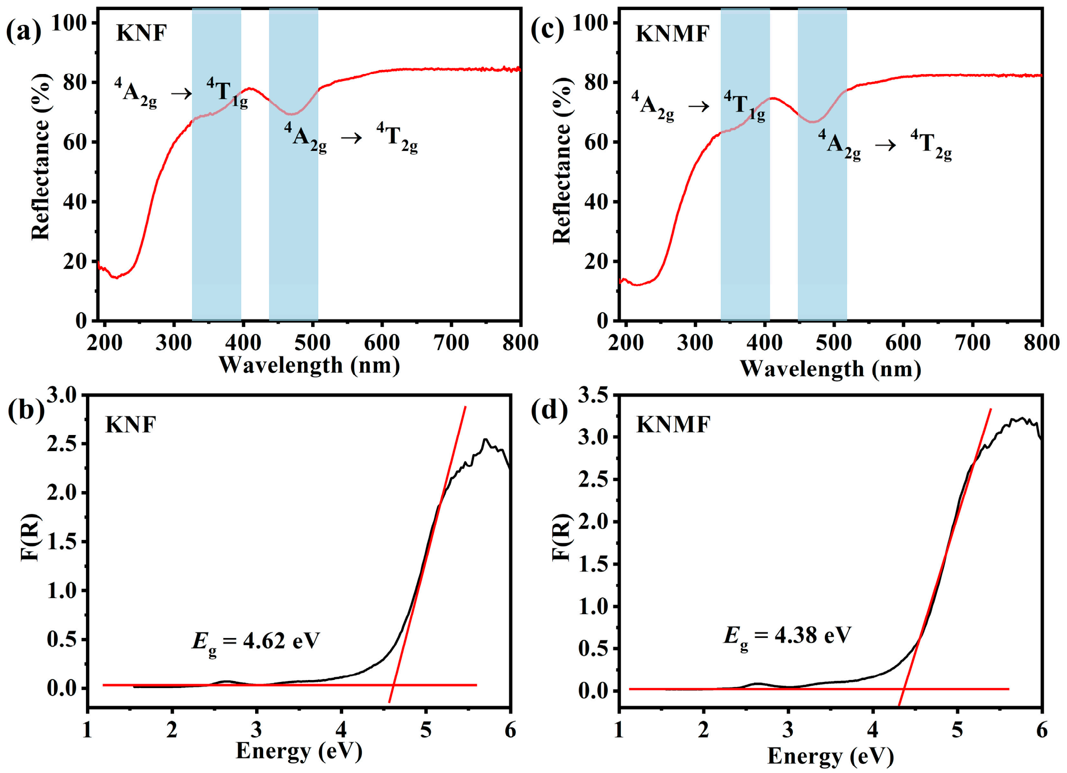

The Tauc relation and Kubelka-Munk relation were used to acquire the band gap from the diffuse reflection spectrum. The band gap can be calculated on the basis of the Tauc relation:

$$[F(R_\infty h\nu)]^n = A(h\nu - E_g) \quad (1)$$

where hν, A and E_g are the energy of photon, proportional constant and the value of the band gap, respectively. n = 2 or 1/2 correspond to a direct transition or indirect transition. And $F(R_\infty)$ is the Kubelka-Munk function, which is represented as follows:

$$F(R_\infty) = \frac{(1-R)^2}{2R} = \frac{K}{S} \quad (2)$$

where R is the reflection, K is the absorption and S is the scattering coefficient [1]. From the linear extrapolation $[F(R_\infty h\nu)]^2 = 0$ in Figure S 2b and d (see red lines), the E_g value for KNF and KNMF are estimated to be 4.62 eV and 4.38 eV, respectively. It is obvious that the E_g value distinctly decreased after the Mo^{6+} ions being doped into the K_2NbF_7:Mn^{4+} phosphor.

Figure S3. Quantum yields of K_2NbF_7:Mn^{4+} (KNF) (a) and $K_2Nb_{1-x}Mo_xF_7$: Mn^{4+} with x = 0.05 (KNMF) (b).

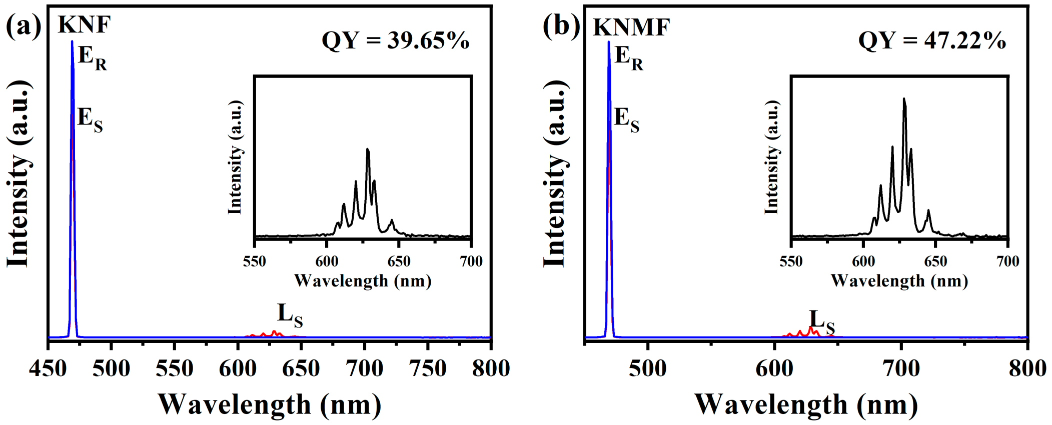

Figure S4. Schematic diagram of the preparation process for K_2MnF_6 and $K_2Nb_{1-x}Mo_xF_7$:Mn^{4+} phosphors.

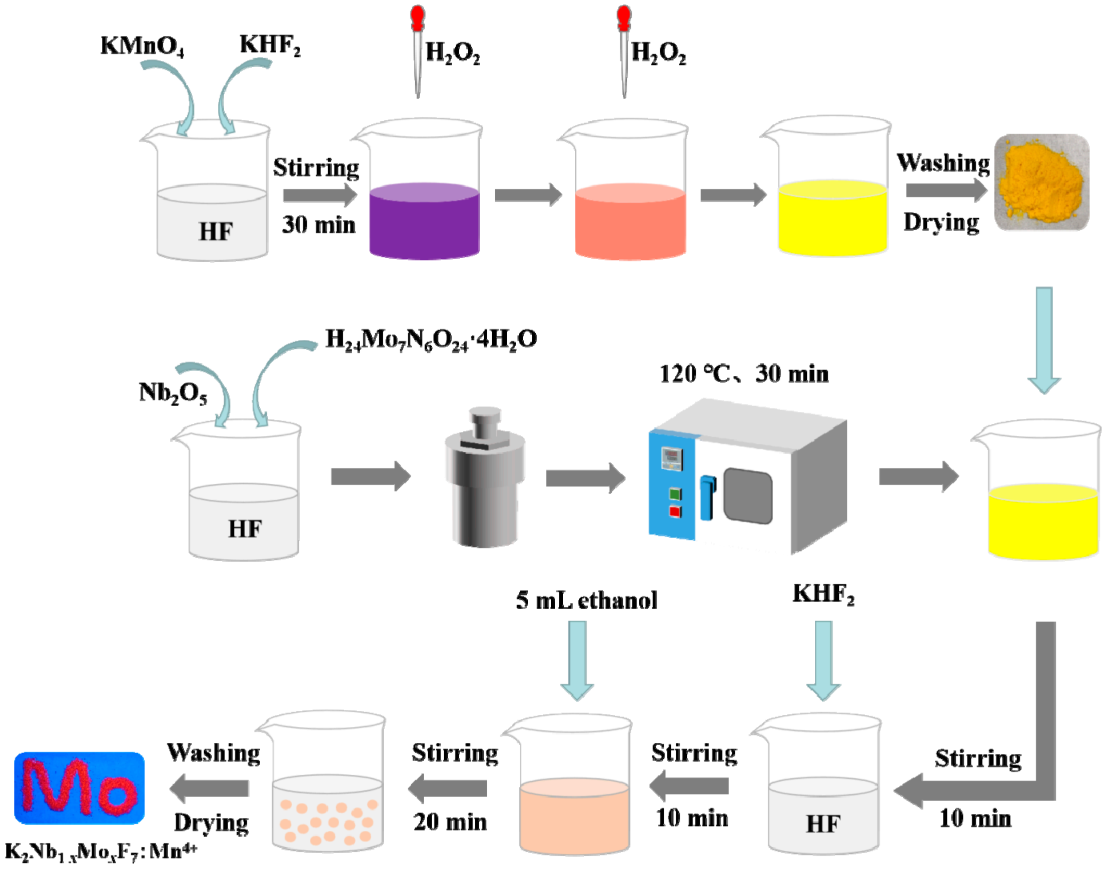

Calculation of the color purity and CCT:

Based on the corresponding chroma coordinates, the relative color temperature and color purity of $K_2Nb_{1-x}Mo_xF_7:Mn^{4+}$ phosphors are calculated by formula (3), (4) and (5), respectively. (x_d, y_d) is the coordinate of the dominant wavelength at 636 nm, and (x_i, y_i) is the coordinate of the CIE 1931 standard source, (x_e = 0.332, y_e = 0.186) expresses the epicenter of convergence [2].

$$\text{Color purity} = \frac{\sqrt{(x_s - x_i)^2 + (y_s - y_i)^2}}{\sqrt{(x_d - x_i)^2 + (y_d - y_i)^2}} \times 100\% \tag{3}$$

$$\text{CCT} = -4493n^3 + 3525n^2 - 6823.3n + 5520.33 \tag{4}$$

$$n = \frac{(x - x_e)}{(y - y_e)} \tag{5}$$

Calculation of the activation energy E_a:

The activation energy E_a is estimated using the Arrhenius formula below:

$$\mathrm{I_T = I_0 / [1 + c * exp(-E_a / kT)]} \tag{6}$$

The formula can be simplified to the following logarithmic equation:

$$\mathrm{ln[(I_0 / I_T) - 1] = -E_a / kT} \tag{7}$$

where I_0 and I_T is the emission intensity at initial and T temperature, respectively, and k is the Boltzman constant.

Table S1. Fitting parameters of decay lifetimes for the $K_2Nb_{1-x}Mo_xF_7$: Mn^{4+} phosphors.

Samples	A_1	τ_1 (ms)	A_2	τ_2 (ms)	τ (ms)	Adj. R-Sqare
x = 0	1.3262	1.5325	1.6873	3.7239	3.1883	0.99341
x = 5%	2.1432	0.1499	1.984	3.6404	3.4917	0.99353
x = 15%	8.3017	0.9335	1.7791	3.6092	2.1459	0.99336

References

[1] Jansen, T.; Baur, F.; Jüstel, T., Red emitting $K_2NbF_7:Mn^{4+}$ and $K_2TaF_7:Mn^{4+}$ for warm-white LED applications. *Journal of Luminescence* **2017,** 192, 644-652.

[2] Hong, F.; Pang, G.; Diao, L.; Fu, Z.; Liu, G.; Dong, X.; Yu, W.; Wang, J., Local structure modulation of Mn^{4+}-doped $Na_2Si_{1-y}Ge_yF_6$ red phosphors for enhancement of emission intensity, moisture resistance, thermal stability and application in warm pc-WLEDs. *Dalton transactions* **2020,** 49, (39), 13805-13817.
